# Optimal statistical arbitrage trading of Berkshire Hathaway stock and its replicating portfolio

**DOI:** 10.1371/journal.pone.0244541

**Published:** 2021-01-15

**Authors:** An-Sing Chen, Che-Ming Yang

**Affiliations:** 1 Department of Finance, National Chung Cheng University, Chia-Yi, Taiwan, R.O.C; 2 School of Accounting and Finance, Xiamen University Tan Kah Kee College, Fujian, P.R.C; Universidad Veracruzana, MEXICO

## Abstract

In this paper, we make use of the replicating asset for statistical arbitrage trading, where the replicating asset is constructed by a portfolio that mimics the returns from a factor model. Using the replicating asset in the context of statistical arbitrage has never been done before in the literature. A novel optimal statistical arbitrage trading model is applied, and we derive the average transaction length and return for the Berkshire A stock and its replicating asset. The results show that the statistical arbitrage method proposed by Bertram (2010) is profitable by using the replicating asset. We also compute the average returns under different transaction costs. For the statistical arbitrage using the replicating asset of the factor model, average annual returns were at least 33%. Robustness is examined with the S&P500. Our results can provide hedge fund managers with a new technique for conducting statistical arbitrage.

## 1. Introduction

Pairs trading is a statistical arbitrage concept, and it has two types: one is the statistical arbitrage, and the other is risk arbitrage. It usually involves using two related securities in the same industry or with similar characteristics and is entered into when their prices deviate from the equilibrium state. The application of pairs trading can be roughly divided into three main steps. First, look for two securities whose prices have the same trend in a given period. Second, observe the change in the spread between the two securities during the subsequent trading period. Third, if the two securities have an equilibrium relationship, a long (short) neutral portfolio can be constructed. When the spread of the two securities reverts to its historical mean, the position is reversed. Existing research finds that pairs trading is related to concepts, such as cointegration, correlation of stock prices, mean regression, overreaction, contrarian selection, and price trends [1–7]. It is undeniable that the effectiveness of pairs trading mainly depends on the modeling and prediction of the spread time series of the two assets (securities, index, or commodities).

If investors want to get the maximum profits, they should look for the spread of pairs with high variance and strong mean reversion. The usual method is to construct a stationary, mean-reverting synthetic asset as a linear combination of securities [8]. However, some researchers argue that this method has little help for forecasting [9, 10]. In light of this, the statistical arbitrage method is developed. For example, pairs trading and its generalizations rely on the construction of mean-reverting spreads, but the mean-reverting spreads must have a certain degree of predictability. So, researchers have developed loss protection methods for pairs trading [11] or used algorithms to estimate trade duration and find optimal preset boundaries [12]. Pairs trading is a simple concept. If the two related stocks' spread expands and diverges from equilibrium, investors can short the high priced stock and buy the low price stock; the investor will profit when the spread converges back towards equilibrium. The concept of pairs trading can be applied to any equilibrium relationship of financial markets or to a portfolio of securities some held short, and the others held long. It uses statistical methods to identify two related stocks and then exploits the potential short term relative mispricing between them and finds all possible portfolios.

In statistical arbitrage trading, investors calculate the historical distance between the standardized daily return paths and choose the pair with the smallest trading distance. If the future price is similar to the past, the spread price may converge again, resulting in a positive return in the zero-cost portfolio. Investors can maximize profits by shorting the overpriced and buying the underpriced. However, this method still has some problems, such as when to trade to maximize the profit of paired trading. Bertram [13] uses the statistical arbitrage trading based on (the Ornstein Uhlenbeck process) to drive the timing of pairs trading entry and exits. Cummins and Bucca [14] followed Bertram’s method and achieved good results. Do and Faff [9, 10] examine the impact of trading costs on pairs trading profitability. For statistical arbitrage, issues such as when, how, and the impact of transaction costs are important.

The relationship between risk and return has always been a worrisome topic in academia and application. Fama and French's three-factor model is designed to capture the relation between average return and size and the price ratios [15]. The three-factor model significantly improved CAPM because it adjusted for the outperformance tendency of strategies based on the additional factors. Although the three-factor model can explain most of the stock returns, there are still researchers who believe that it is not complete [16–18]. Carhart [16] added the momentum factor and proposed a four-factor model. Titman et al. [17] argue that increasing capital investments subsequently leads to negative benchmark-adjusted returns. Novy-Marx [18] measures profitability using the gross profits-to-assets and shows that it provides approximately the same power as book-to-market in forecasting the cross-section of average returns. The five-factor model added the factors of profitability and investment; the evidence showed that the three-factor model was insufficient for explaining expect return because it ignored a lot of the variation in average returns related to profitability and investment [19]. The empirical tests of the five-factor model use the average returns on portfolios formed on size, B/M (book to market ratio), profitability, and investment. Recently, newer factor models, such as BAB and QMJ, are proposed [20, 21]. Frazzini et al. [22] studied Warren Buffett's company, Berkshire Hathaway's profitability, and performance; they propose several quantifiable factors to analyze its performance. They refer to the papers by Carhart [16], Frazzini and Pedersen [20], and Asness et al. [21] to propose an alternative factor model. In this study, we will refer to it as the "Buffet-factor model." We present analytic formulae and solutions for calculating optimal statistical arbitrage strategies with transaction costs using the Buffet-factor model to form the replicating portfolio. We assume that the synthetic asset formed by the Berkshire Hathaway stock and its replicating portfolio can be described by the Ornstein Uhlenbeck process. Our main results show that the replicating portfolio can be effectively paired with the original asset in a pairs trading statistical arbitrage framework and verify that this method is rewarded.

In past literature, most research explores the arbitrage of two similar nature assets. There does not exist research on using the factor model to create replicating assets for arbitrage. The main contribution of this paper is using the factor model to form a replicating asset and then constructing the synthetic asset with other assets for statistical arbitrage. Methodologically, we form the replicating asset (portfolio) by using the Buffett- and five-factor model following the method described in Asness et al. [21]. We verify that this method can indeed create replicating assets that exhibit similar properties to Berkshire A stock and that the replicating asset can be paired with the original Berkshire A stock for statistical arbitrage, profitably. To implement the statistical arbitrage, we refer to the findings of Bertram [13] and apply them to our experiments. In particular, we use the Buffet-, five-factor model, and the Ornstein Uhlenbeck process to perform statistical arbitrage for the Berkshire A stock and the S&P 500 portfolio. The resulting analysis provides the mathematical framework which can be used to explore the relationships between the replicating portfolio and Berkshire's stock and offer insight into the dynamics of trading strategies. We use the Ornstein Uhlenbeck process to build a continuous trading strategy for the original asset and its replicating portfolio and compute the trade length and the return of the strategy based on the transit time of the process. The results of this paper show that Berkshire A paired with its replicating portfolio provides returns of at least 33% under statistical arbitrage and S&P500 at least 4.8%.

The remainder of the paper is organized as follows. Section 2 discusses the literature related to statistical arbitrage and factor models. Section 3 presents the data and statistical arbitrage trading model of the Ornstein Uhlenbeck process. Section 4 presents the results of the empirical analysis and examines robustness to varying transaction costs. Section 5 concludes and summarizes the main results of the paper.

## 2. Related literature

### Literature on statistical arbitrage

The most common form of pairs trading involves forming a portfolio of two related stocks whose relative pricing departs from its ‘equilibrium.’ The equilibrium may involve the concepts of cointegration, mean regression, overreaction, and reversal strategies. The success of statistical arbitrage depends on finding two suitable securities, then modeling and forecasting of spread time series. One popular method is the distance approach, which explores different dimensions and implications of pairs trading strategies, such as accounting information, news, liquidity, sensitivity, transaction costs, etc. [8–10, 23–25].

Jegadeesh and Titman [5] find the strategies that purchase stocks that have outperformed in the past, and short stocks that have underperformed in the past produce significantly positive returns. They argue that the profitability of these strategies is not due to their systematic risk or delayed stock price reactions to common factors. Gatev et al. [8] first proposed the distance approach. They use all U.S. stocks from the CRSP daily dataset from 1962 to 2002. They design a simple algorithm for choosing pairs and test the profitability of several straightforward, self-financing trading rules. They calculate the sum of Euclidean squared distance (SSD) for n stocks and choose the smallest SSD to construct a portfolio. They then choose to enter the transaction when the price of the paired asset is greater than two standard deviations, and when the price matched the average price, they sell the paired asset. They find average annualized excess returns of about 11% for the top pairs portfolios and that the profits do not appear to be caused by simple mean reversion. However, Do and Faff [9] apply the Gatev et al. [8] methodology with more recent data and find the profit show a declining trend when the naive trading rule is used. Do and Faff [10] modify the 2010 paper on the U.S. CRSP stock but extend the sample period from 1963 to 2009. In the newer study, they considered transaction costs and allowed securities to be matched across 48 Fama-French industries. This restriction created more meaningful pairs, because all sample companies are now matched within the same sectors. Their results show that the pairs trading strategy remains profitable, albeit at much more modest levels. Cohen and Frazzini [26] find substantial customer-supplier links in the U.S. stock market that allow for return predictability in the context of this strategy.

Another statistical arbitrage concept is cointegration. The method of cointegration describes as follows: first, choose two cointegrated stock price series; second, construct a long (short) position when stocks deviate from their long-term equilibrium; finally, close the position after convergence or at the end of the trading period [27–30]. Hong and Susmel [31] implement the cointegration approach to common stocks. They analyze pairs-trading strategies for 64 Asian shares listed in their local markets and listed in the US as ADRs from 1991 to 2000. They assume these pairs to be cointegrated but provide no test results in their paper. Vidyamurthy [29] uses the Arbitrage Pricing Theory (APT) to identify stocks with similar common return components. They developed a framework for forecasting using the cointegration method and analysis the mean reversion of the residuals. Under the multivariate cointegration approach, Dunis and Ho [32] use cointegration relationships to construct index-tracking portfolios for the EuroStoxx 50 index. They then take different subsets of the index constituents and estimate the joint cointegration vector for these constituents and the EuroStoxx 50 index. They find that the tracking baskets have an outperformance versus the benchmark in terms of absolute returns and Sharpe ratio. Caldeira and Moura [33] apply the univariate cointegration approach to the 50 most liquid stocks of the Brazilian stock index Bovespa. They show that this strategy generates excess returns of 16.38% per year. Moreover, it also gives a Sharpe Ratio of 1.34 and a low correlation with the market.

Mean reversion is key to pairs trading. The stochastic spread method models the mean-reverting process of pairs trading as an Ornstein Uhlenbeck process. Elliott et al. [34] advance a mean-reverting Gaussian Markov chain model for the spread, which is observed in Gaussian noise. They compare the model with subsequent observations of the spread to determine appropriate investment decisions. They believe this approach can be applied to any financial market to gain wealth, even though it is at times out of equilibrium. Most financial information is non-Stationary and non-Gaussian. Therefore, it is necessary to deal with the stability of the time series data. The Ornstein Uhlenbeck process is a stationary Gauss Markov process, and is homogeneous in time. This process has a long-term mean and exhibit mean reversion. The process can be viewed a modification of random walk in continuous time or Wiener process. The Ornstein Uhlenbeck process can be considered as the continuous-time analog of the AR (1) process. Because the Ornstein Uhlenbeck process is static, the return is deterministic.

Bertram [13] derives the entry/exit time and analytical formula for the trading thresholds for synthetic assets formed by pairs, whose price assumptions follow the Ornstein Uhlenbeck process. He showed that the optimal thresholds were symmetric around the mean both for maximizing the return per unit time and the Sharpe ratio. His result also provides the optimal entry and exit points for arbitrage trading at a given transaction cost. Cummins and Bucca [14] believe a rational investor would aim for a high-profit opportunity. So, the rational pairs trade should have a combination of the lowest drift in spread mean and highest spread variance features. They apply Bertram’s method to the spread trading for crude oil and refined products markets. Their result showed evidence of aggregate upward and downward mean reversion, and profitable strategies with Sharpe ratios of greater than two. Their research shows that Bertram's method has profitability potential for non-Gaussian processes. Avellaneda and Lee [35] and D'Aspremont [36] developed a method of synthetic mean-reverting portfolios, which uses the concept of Elliott et al. [34]. They use canonical correlation analysis to construct mean-reverting portfolios with a limited number of assets. Their result shows that the systematic component of stock returns explains between 40 and 60% of the variance. Huck and Afawubo [37] explore the performance of a pairs trading system based on various pairs-selection methods. They use the components of the S&P 500 index as an observation target. They argue that when the stock price deviates from equilibrium, the investor can enter the trade (long or short) after controlling for risk and transaction costs. They also show that the distance method generates insignificant excess returns, but the cointegration method provides a high, stable, and robust return.

### Literature on the five-factor and Buffett-factor model

Under the capital asset pricing model (CAPM), the expected return of a stock is related to its Beta coefficient. However, this model has difficulties in explaining actual stock market returns. First, its assumptions are difficult to maintain in the actual capital market. Second, the factors affecting expected returns may be more than one. The Arbitrage Pricing Theory (APT) is an extension of CAPM. The pricing model given by both the APT and the CAPM are models under equilibrium. The difference is that the APT model is based on the concept of arbitrage. If the market does not reach equilibrium, there will be risk-free arbitrage opportunities in the market. The APT model uses several factors to explain the expected return on risky assets, and according to the no-arbitrage principle, there is an approximately linear relationship between the equilibrium return of risk assets and the risk factors.

Fama and French [15] find that companies with smaller market value and higher book value/ market value ratio are more likely to achieve an average rate of return above market levels. So, they join the size factor (SMB) and value factor (HML) with the original CAPM. The resulting model is the well-known three-factor model. This model can explain about 90% of stock returns. Carhart [16] uses a four-factor model, which includes the market factor, size factor, value factor, and momentum factor, to control the impact of systemic risk on stocks. His results show that the four-factor model is an improvement over the Fama-French's three-factor model and was able to explain the short‐term persistence in equity mutual fund returns. Titman et al. [17] find the firms that substantially increase capital investments subsequently achieve negative benchmark-adjusted returns. The negative abnormal capital investment/return relation is shown to be stronger for firms that have more considerable investment discretion, i.e., firms with higher cash flows and lower debt ratios. Novy-Marx [18] identifies a proxy for expected profitability that is strongly related to the average return. His results show that gross profits-to-assets has the same power as book-to-market in predicting the cross-section of average returns. Also, profitable firms produce significantly higher returns than unprofitable firms, despite having significantly higher valuation ratios. Fama and French [19] added the factors of profitability and investment to the three-factors model and showed that the five-factor model explains between 71% and 94% of the cross-section variance of expected returns on the portfolios.

Asness et al. [21] define 'quality' security as one that has characteristics that an investor is willing to pay a higher price for, namely, stocks that are safe, profitable, growing, and well managed. They present a quality-minus-junk (QMJ) factor to describe these characteristics, and they find that stocks sorted on this factor have significant risk-adjusted returns in the U.S., and globally across 24 countries. The QMJ factor is constructed by a portfolio that longs high-quality stocks and shorts low-quality stocks. Frazzini and Pedersen [20] discuss a market-neutral betting-against-beta (BAB) factor, which is constructed by a portfolio that longs leveraged low-beta assets and shorts high-beta assets. They find two key results: (1) high beta is associated with low alpha (2) stocks sorted on the BAB factor produces significant positive risk-adjusted returns.

## 3. Data and methodology

In this paper, we examine the statistical arbitrage trading between the Berkshire Hathaway stock and its replicating portfolio. For clarity of exposition, in the remainder of this paper the term “replicating portfolio” will be replaced with the term “replicating asset” where appropriate for increased clarity. The empirical tests of this study consist of two parts. First, we utilize the five-factor model and Buffett-factor model to construct the replicating asset. Second, we use the Ornstein Uhlenbeck process to test the statistical arbitrage trading of the synthetic asset constructed from the original Berkshire Hathaway stock and its replicating asset.

For this study, we utilize the factors data of the five-factor and the Buffett-factor model and the price of Berkshire Hathaway stock. The various factors of the models are obtained from the Kenneth R. French data library and the AQR data library. Berkshire Hathaway’s stock return and price data are from the CRSP database. Berkshire Hathaway’s stock has two classes: class A and class B. Berkshire Hathaway introduced share class B in April 1996. For this study, focus on Class A stocks based on the longer length of the data. We use daily data from 1980/3/17 to 2018/09/28. We use the five-factor model, and the Buffett-factor model to construct the replicating asset for Berkshire Hathaway class A stock. We denote it with BerkA*. We then use the replicating asset and the original Berkshire Hathaway stock to conduct the statistical arbitrage experiments. The data period used in this study spans from 1980/03/17 to 2018/09/28, which gives a total of 9720 daily observations.

Fama and French [15] design the three-factor model to capture the relation between average return and Size (market capitalization, computed as the price times shares outstanding) and the relation between average return and price ratios like B/M. However, Titman et al. [17] and Novy-Marx [18] note that the three-factor model misses much of the variation in average returns associated with profitability and investment. The three-factor model is incomplete. Fama and French [19] add profitability and investment factors to the three-factor model. The five-factor model is presented as follows:

$$R_{it}-R_{ft}=a_{i}+b_{i}(R_{mt}-R_{ft})+s_{i}{SMB}_{t}+h_{i}{HML}_{t}+r_{i}{RMW}_{t}+c_{i}{CMA}_{t}+e_{it}$$ (1)

A five-factor model that captures the size, value, profitability, and investment patterns in average stock returns can perform better than the three-factor model. In the five-factor model, *R*_*it*_ is the return on security or portfolio *i* for period *t*, *R*_*ft*_ is the risk-free return, *R*_*mt*_ is the return on the value-weight (VW) market portfolio. The size factor *SMB*_*t*_ is the return from a diversified portfolio of small stocks less the return on a diversified portfolio of large stocks. A positive loading on SMB reflects a tendency to buy small stocks [15], but Berkshire’s negative loading reflects a tendency to buy large stocks [22]. The value factor *HML*_*t*_ is the difference between the returns on diversified portfolios of high and low B/M stocks. Berkshire stock shows a positive tilt, which reflects a tendency of buying stocks that have a high book value relative to their market value [22]. The profitability factor *RMW*_*t*_ is the difference between the returns on diversified portfolios of stocks with robust and weak profitability. The investment factor *CMA*_*t*_ is the difference between the returns on diversified portfolios of the stocks of low and high investment firms, and *e*_*it*_ is a zero-mean residual.

Buffett’s factor model was proposed by Frazzini et al. [22]. They show that Berkshire Hathaway’s performance can be explained mainly by exposures to value, low-risk, and quality factors. It has been documented that value stocks have higher average returns than growth stocks [38, 39], and high-quality stocks outperform than junk stocks on average [21]. Among the traded U.S. stocks, Berkshire Hathaway has one of the highest Sharpe ratios for more than 30 years. Frazzini et al. [22] identify several features of Buffett's portfolio: “safe” (low volatility and low beta), “cheap” (i.e., stocks with low price-to-book ratios (value stocks), and high-quality (stocks that are profitable, stable, growing, and have high payout ratios). However, the stocks exhibit these characteristics usually perform well, not only the stock that Buffett bought. These well-known factors seem not fully capture the performance of Berkshire stock.

Carhart [16] constructed a four-factor model by combining the three-factor model of Fama and French [15] and the momentum effects of Jegadeesh and Titman [5]. The momentum factor UMD is constructed by buying the stocks that outperform the market and shorting the stocks that underperform. UMD is insignificant to Berkshire stock. Frazzini et al. [22] expanded the four-factor model and added the equity factor (Betting Against Beta (BAB)) of Frazzini and Pedersen [20] as well as the quality factor (Quality Minus Junk (QMJ)) of Asness et al. [21]. The BAB factor is constructed by a portfolio that longs low-beta assets, leveraged to a beta of one: and shorts high-beta assets, de-leveraged to a beta of one. The QMJ factor is constructed by a portfolio that longs high-quality stocks and shorts low-quality stocks. The Buffett-factor model of Frazzini et al. [22] as follows:

$$R_{it}-R_{ft}=a_{i}+m_{i}{MKT}_{t}+s_{i}{SMB}_{t}+h_{i}{HML}_{t}+u_{i}{UMD}_{t}+b_{i}{BAB}_{t}+q_{i}{QMJ}_{t}+e_{it}$$ (2)

We modify the equation of Carhart's [16] and add the factors of BAB and QMJ and let its features be consistent with the five-factor model. The equation is specified as follows:

$$R_{it}-R_{ft}=a_{i}+m_{i}(R_{mt}-R_{ft})+s_{i}{SMB}_{t}+h_{i}{HML}_{t}+u_{i}{UMD}_{t}+b_{i}{BAB}_{t}+q_{i}{QMJ}_{t}+e_{it}$$ (3)

This section provides the mathematical exposition of the Ornstein Uhlenbeck process statistical arbitrage trading model of Bertram [13]. Under this model, a continuous trading strategy is formed by a series of separate transactions executed on a continuous-time stochastic process. Thus, many trading strategies are a function of the frequency which these transactions occur. The trading frequency is defined by how many times the strategy trades per unit time. So, we can model the price of the traded security *P*_*t*_ as,

$$P_{t}=e^{x_{t}};\;X_{t0}=x_{0}$$ (4)

*X*_*t*_ denotes the synthetic asset. For our experiments we define *X*_*t*_ = log(*Berkshire stock price*)−log(*replicating asset price*). Since the replicating asset is constructed using the theoretically accurate asset pricing models of Eqs (2) and (3); *X*_*t*_, the spread between two asset log price series should follow a zero-mean Ornstein Uhlenbeck process. An Ornstein Uhlenbeck process is symmetric. Mathematically, *X*_*t*_ can be written as the following stochastic differential equation,

$${dX}_{t}=-\alpha X_{t}dt+\eta dW_{t}$$ (5)

where α and η > 0, and *W*_*t*_ is a Wiener process. Suppose *T* is the complete trading period. Define the entry levels of the trading strategy by *X*_*t*_ = *a* and exiting the trade at *X*_*t*_ = *m*. We assume that *a < m*, this implies that *a <* 0 and *m* = −*a*. A complete trading cycle time is presented as follows:

$$T=T_{a\to m}+T_{m\to a}$$ (6)

Let *r*(*a*,*m*,*c*) denote the return per trade as a function of *a*,*m*, and transaction cost. Given that the *X*_*t*_ represents the log price, the continuously compound rate of return for a single trade after accounting for transaction cost can be written as *r*(*a*,*m*,*c*) = (*m*−*a*−*c*). In a profitable strategy, the return must exceed the transaction costs from entry moving to exit. Bertram [13] shows that under these conditions, the expected value per unit time and the variance of the return per unit time for the strategy can be written as,

μ(a,m,c,t)=r(a,m,c)/E(T) (7)

$$\sigma^{2}(a,m,c,t)={r(a,m,c)}^{2}V(T)/{E(T)}^{3}$$ (8)

where *E*(*T*) and V(*T*) are the mean and variance of T. Even though the process *X*_*t*_ is stationary, the return of every trade is stochastic; the time frame for return realization is random. A trade may take a long time before reaching the exit level and have a significant deviation away from the exit level during the time frame. We can use the first pass time of the Ornstein Uhlenbeck process to calculate the expression of the strategy return and variance. Bertram [13] assumes *a*<*m*, *a*<0, and −*a* = *m*. *T*_*a*→*m*_ represents the time to transition from *a* to *m*, and *T*_*m*→*a*_ is the time to transition from *m* to *a*, and the independence of the two times follows from the Markovian property of the Ornstein Uhlenbeck process. The maximum expected return is then given as follows:

$$\mu^{*}(a,c)=\frac{\alpha (2a+c)}{2\pi Erfi(a\sqrt{\alpha }/\eta )}$$ (9)

and the optimal value of *a* will satisfy the following equation:

$$\begin{array}{l} a=-\frac{c}{4}-\frac{c^{2}\alpha }{{4(c^{3}\alpha^{3}+24c\alpha^{2}\eta^{2}-4\sqrt{3c^{4}\alpha^{5}\eta^{2}+36c^{2}\alpha^{4}\eta^{4}})}^{\frac{1}{3}}}\\ \;-\frac{{\left(c^{3}\alpha^{3}+24c\alpha^{2}\eta^{2}-4\sqrt{3c^{4}\alpha^{5}\eta^{2}+36c^{2}\alpha^{4}\eta^{4}}\right)}^{1/3}}{4\alpha } \end{array}$$ (10)

## 4. Results

This section presents the results of optimal statistical arbitrage trading of Berkshire Hathaway stock with its replicating asset. First, we construct a replicating asset, which will have similar risk and return characteristics with the actual Berkshire A stock price by using the five-factor model (Eq (1)) and the Buffett-factor model (Eq (3)). The factor loadings of the replicating asset are estimated by regressing the excess return of Berkshire A on right-hand-side factors of Eq (1) and Eq (3). Specifically the excess return of Berkshire A is the as the dependent variable and the factors on the right-hand-side of Eq (1) and Eq (3) are the independent variables. The estimated coefficients are then used as the portfolio weights for the construction of the replicating asset. The returns of the replicating portfolio will, in the long run, match the returns of the Berkshire A stock, since the replicating portfolio is constructed from theoretically correct asset pricing model specifications. In the remainder of our paper, we will denote the replicating portfolios as simply Buffett- or five-factor model. Second, we use the replicating portfolios as input to the pair trading simulation tests. The five- or Buffet-factor models capture most of the factors affecting stock price return. Assuming the investor is rational, the investor will review past performance and adjust the trading strategy in each fixed period. Therefore, in our experiments, we model this behavior by assuming that investors will close their open positions (take losses or capitalize the gains) the last trading day of each year and reset their positions the first trading day of the next year using the information up to that day. This reset will prevent the prices of the two paired assets (Berkshire A stock and the replicating asset) from drifting too far apart. Specifically, at the beginning of each year, investors will recalculate the theoretical value of the replicating asset and compare it with the market value of Berkshire A stock and take the appropriate positions for the next cycle (short the overpriced asset and long the underpriced asset).

**Fig 1:**
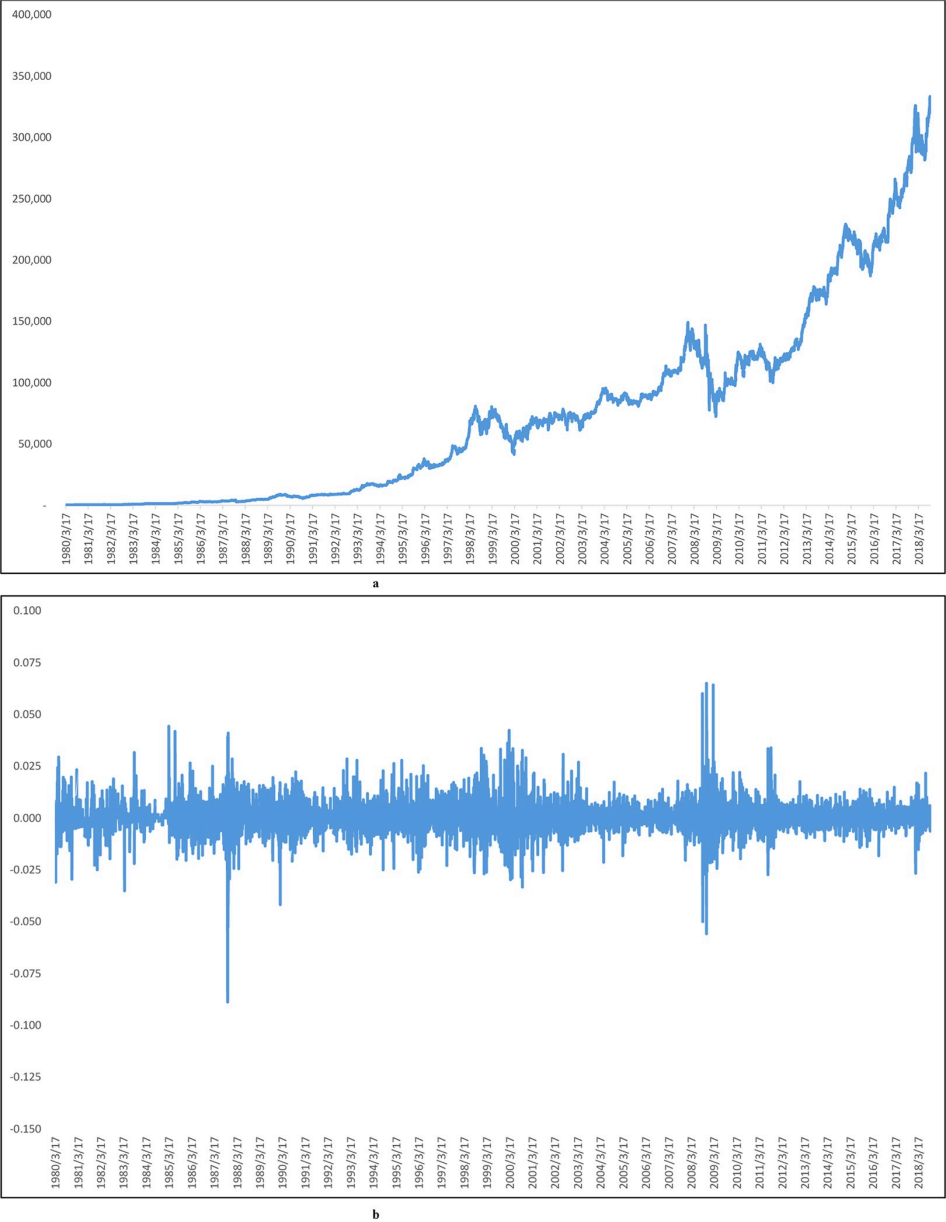
a: The trend of Berkshire A stock price, b: Berkshire A stock price returns. Fig 1A shows Berkshire A stock price trend from 1980/3/17-2018/09/28. The figure shows that Berkshire A stock price rises very quickly. It also shows that the return volatility for Berkshire A is also relatively high. Fig 1A shows that the stock price fell significantly during the period of the recent financial crisis, but the stock price also rises faster after the financial crisis. Fig 1B shows the return of Berkshire A stock price from 1980/3/17-2018/09/28. Fig 1B shows that the volatility of stock price return was significantly higher in 1987 and 2008 than in other periods.

Fig 1A and 1B show the stock price trends and stock price returns of Berkshire Hathaway A from 1980/03/17-2018/09/28. In Fig 1A and 1B, we can find the stock price dropped significantly, and the stock price return also fluctuated significantly more than other times during the financial crisis.

**Fig 2:**
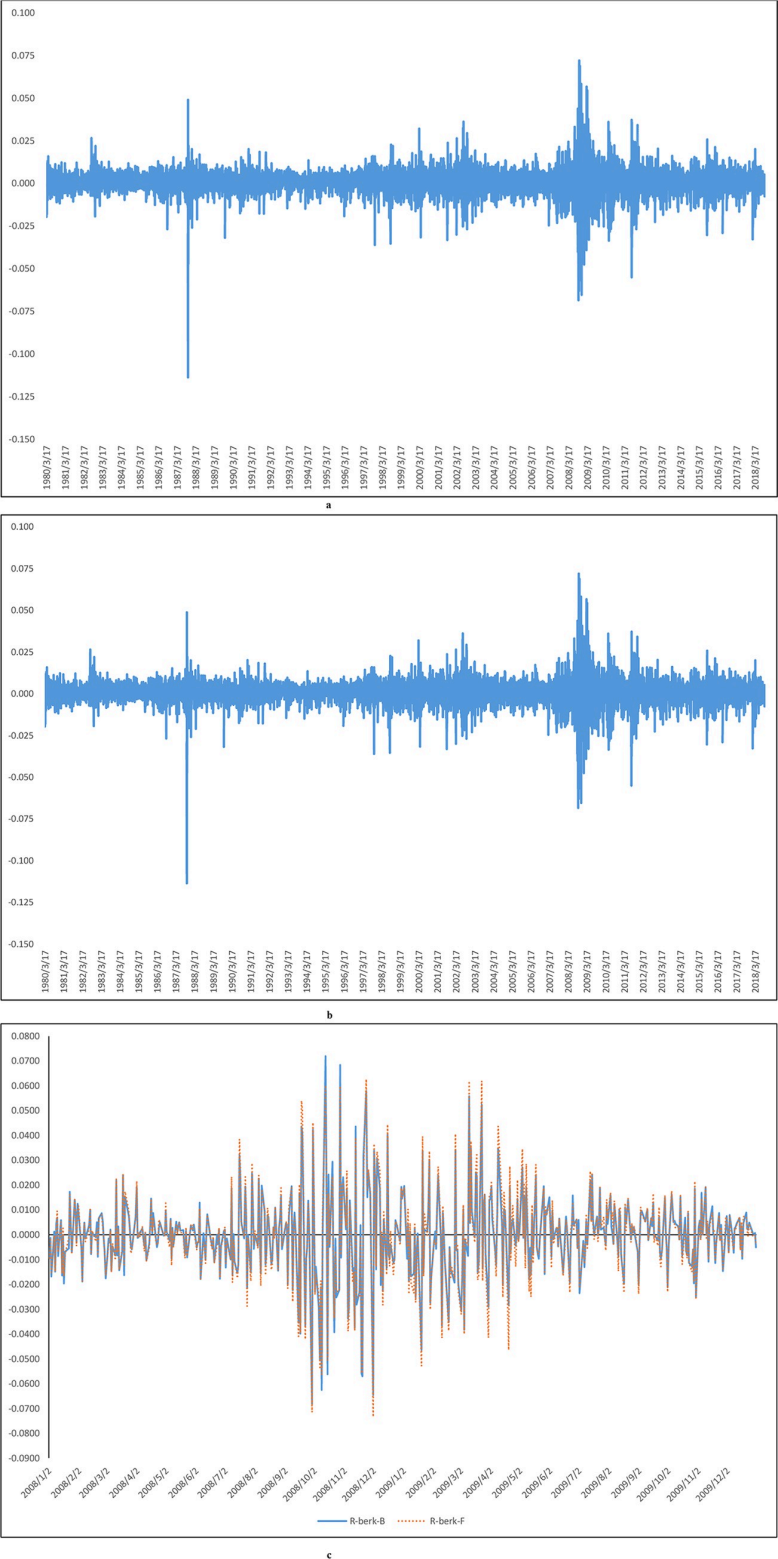
a: Buffett-factor model returns, b: five-factor model returns, c: Buffett-factor model returns and five-factor model returns for the sub-period (2008–2009). Fig 2A shows the returns of the replicating asset constructed by using the Buffett-factor model. Fig 2B shows the returns of the replicating asset constructed by using the five-factor model. We can find that the overall behavior patterns of the two replicating assets are similar to those of the original Berkshire A stock. Fig 2C overlays the information presented in Fig 2A and 2B in one plot using a shorter time frame, 2008–2009. This figure shows that the information presented in Fig 2A and 2B, which depict the returns of the replicating assets obtained from using the Buffett- and five-factor models are not identical to each other. In the figure, the maximum return of the Buffett-factor model is 7.2%; the minimum return is -0.068%. The maximum return of the five-factor model is 6.26%; the minimum return is -0.0733.

In our experiments, we use two different factor models to construct the replicating asset. Fig 2 shows the returns of the replicating assets constructed using the Buffett- and five-factor models. Fig 2A and 2B shows that the overall return pattern of the replicating assets is similar to the return pattern of the original asset in Fig 1B. In the remainder of this paper, we use BerkA* to denote the theoretical stock price of the replicating asset constructed by using the Buffett- or five-factor models.

It should be noted that Fig 2A and 2B, which depict the returns of the replicating assets obtained from using the Buffett- and five-factor models are not identical to each other. They only look similar to each other because the time-period for the two figures is quite long, spanning from 1980/3/17-2018/09/28, which causes the two figures to look very similar to each other. Fig 2C plots the information in Fig 2A and 2B using a shorter period, from 2008–2009 only, and overlay the results of the two models on top of each other using only one graph. Fig 2C shows more clearly that the information presented in Fig 2A and 2B are not identical to each other.

**Fig 3:**
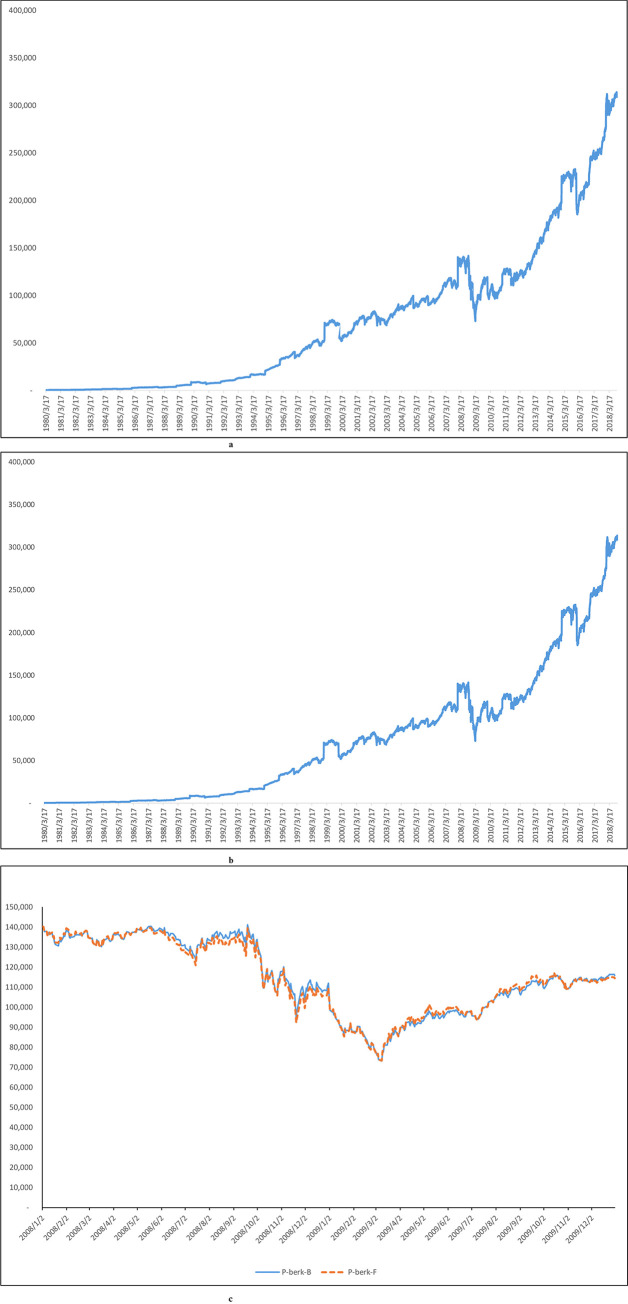
a: Replicating asset price of Buffett-factor model, b: Replicating asset price of five-factor model, c: Replicating asset price of Buffett-factor model and replicating asset price of five-factor model for the sub-period (2008–2009). Fig 3A shows the replicating asset price when the replicating asset is constructed using the Buffet model. Fig 3B shows the replicating asset price when the replicating asset is constructed using the five-factor model. Overall price behavior closely matches the price behavior of the original Berkshire A price. Fig 3C overlays the information presented in Fig 3A and 3B in one plot using a shorter time frame, 2008–2009. This figure shows that the information presented in Fig 3A and 3B, which depict replicating asset price when the replicating asset is constructed using the Buffet- and five-factor models, are not identical to each other. The maximum value of replicating assets price of Buffett- factor model is $141,109; the minimum value is $73,088. The maximum value of the five-factor model is $140,284; the minimum value is $73,375.

Fig 3A shows the price behavior of the replicating asset constructed using the Buffet-factor model. Fig 3B shows the price behavior of the replicating asset constructed using the five-factor model. Figs 3A, 3B and 1A show nearly identical patterns and trends. The figures show that the replicating assets constructed by the Buffet- and five-factor model does an excellent job of replicating the Berkshire stock and thereby can serve as a good opposing asset in pairs trading statistical arbitrage. Fig 3C plots the information in Fig 3A and 3B using a shorter period, from 2008–2009 only, and overlay the results of the two models on top of each other using only one graph. Fig 3C shows that the information presented in Fig 3A and 3B are not identical to each other.

**Fig 4:**
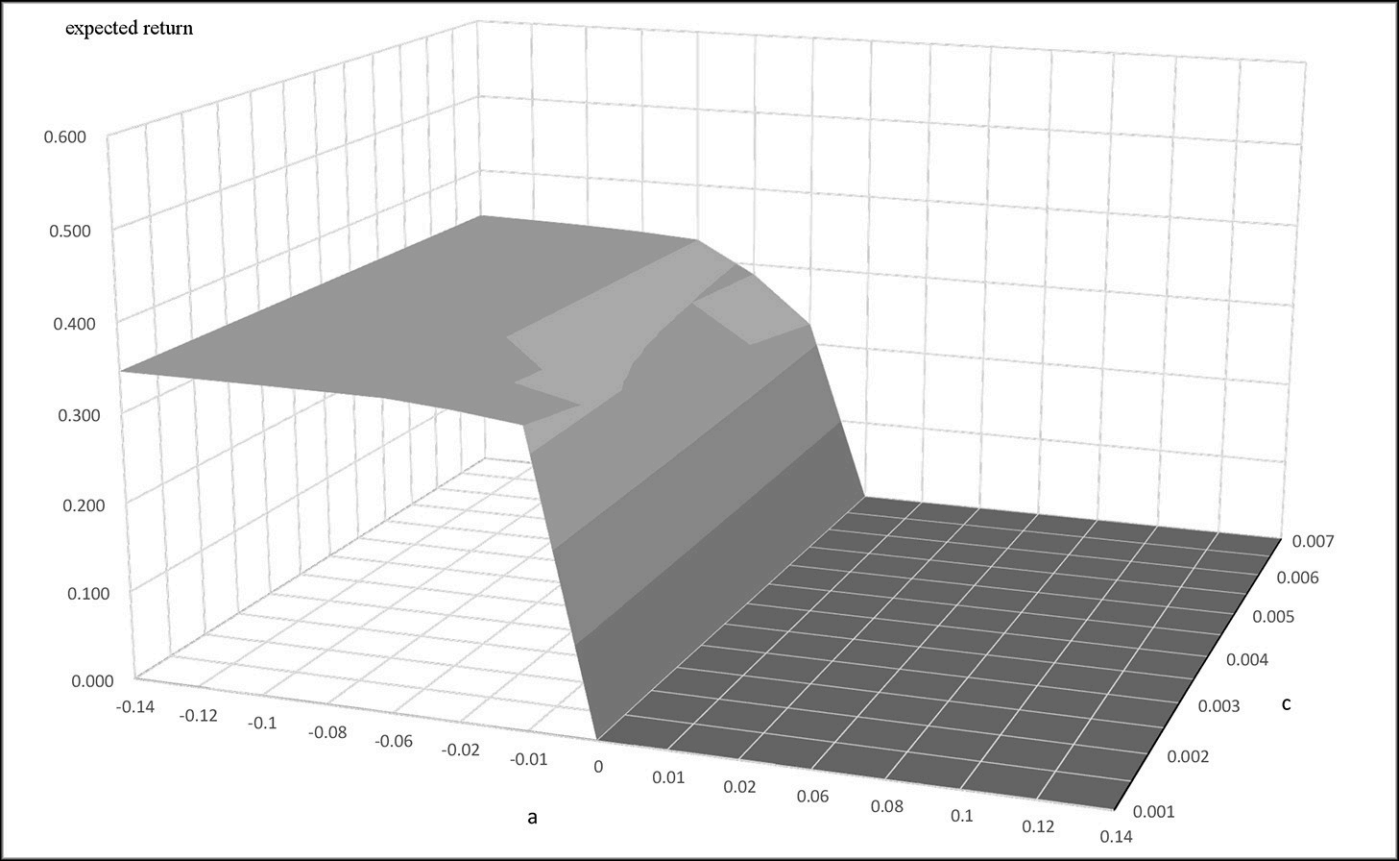
**The relationship of expected return, a, and c when the replicating asset is constructed by the Buffet-factor model.** This figure presents the relationship between the expected return, a, and transaction cost when the replicating asset is constructed by the Buffett-factor model. This example uses parameters α = 0.006439, η = 0.05401, c = 0.001~0.007.

**Fig 5:**
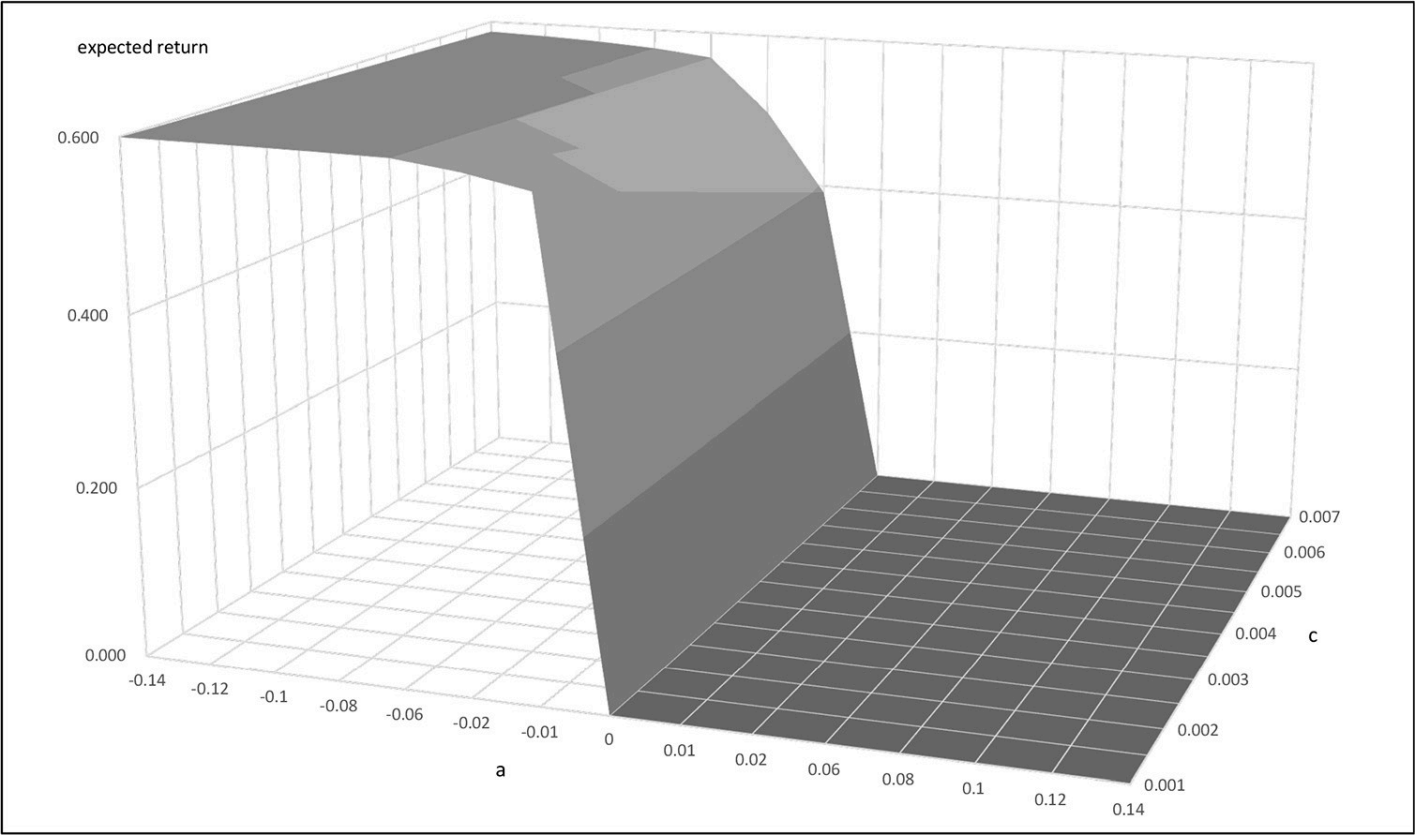
**The relationship of expected return, a, and c when the replicating asset is constructed by the five-factor model.** This figure presents the relationship between the expected return, a, and transaction cost when the replicating asset is constructed by the five-factor model. This example uses parameters α = 0.01702, η = 0.05748, c = 0.001~0.007.

We next apply the Ornstein Uhlenbeck process arbitrage strategies discussed in Bertram [13] and Cummins and Bucca [14] to find the optimal entry and exit points for the initiation and cessation of pairs trading. We get α = 0.006439, η = 0.05401 for Buffett-factor model, and α = 0.01702, η = 0.05748 for five-factor model. Figs 4 and 5 present the results of using the Buffett- and five-factor model as the replicating asset. The figures present the plots of the expected return and entry-level with different transaction costs. They show that the trading frequency and transaction cost has a strong influence on the profitability of the trading strategy. Trading strategies are influenced by "a" and transaction cost to form the expected return bands. In the trading bands, for a given transaction cost, the larger "a" makes the expected return smaller. On the other hand, for a given "a" value, the larger the transaction cost makes the expected return smaller. In sum, the "a" and transaction costs are unfavorable to the expected return of the trading strategy. The equations for the mean and variance of the return allow us to determine the trading bands that optimize the trading strategy. We use Eq (8) to get a, when a<0. The expected return is equal to 0 when a>0.

Table 1 shows the expected returns of using the optimal statistical arbitrage strategies for different transaction costs and "a." The optimal solution for "a" is from Eq (8). In the Table, "c" represents the transaction cost, and "a" represents the entry-level. Table 1 shows that "a" and expected return will become smaller as the transaction costs increase, regardless of whether the replicating asset is constructed using the Buffett- or five-factor model.

**Table 1:** The expected return of optimal statistical arbitrage of Berkshire A and replicating asset pair.

	Buffett-factor model		five-factor model	
c	a	expected return	a	expected return
0.001	-0.06978	0.34515	-0.05274	0.59577
0.002	-0.08841	0.34371	-0.06678	0.59246
0.003	-0.10138	0.34250	-0.07663	0.58970
0.004	-0.11178	0.34142	-0.08452	0.58724
0.005	-0.12059	0.34043	-0.09122	0.58499
0.006	-0.12832	0.33951	-0.09712	0.58289
0.007	-0.13526	0.33865	-0.10241	0.58092

This table presents the expected return from using the optimal arbitrage strategy under the Ornstein Uhlenbeck process of Berkshire A stock and its replicating asset BerkA*. The table lists the expected returns when the replicating asset is constructed using the Buffett- and five-factor model under different transaction costs. The optimal solution for "a" is from Eq (8). For the Buffet-factor model α = 0.006439, η = 0.05401, for the five-factor model α = 0.01702, η = 0.05748. "c" is the transaction cost and "a" represents the entry point.

**Fig 6:**
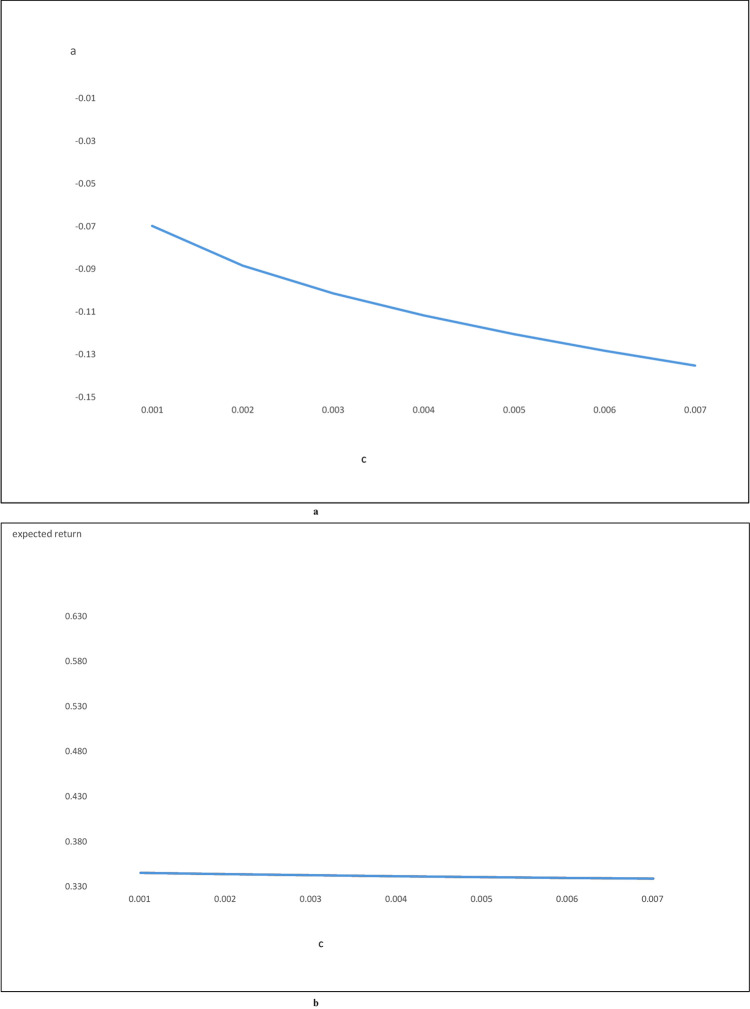
a: c vs a for Buffet-factor model, b: c vs expected return for Buffet-factor model. Fig 6A shows the relationship between transaction cost and Fig 6B shows the relationship between transaction cost and the expected return from the optimal trading strategy. The replicating asset for Fig 6 is constructed using the Buffett-factor model.

**Fig 7:**
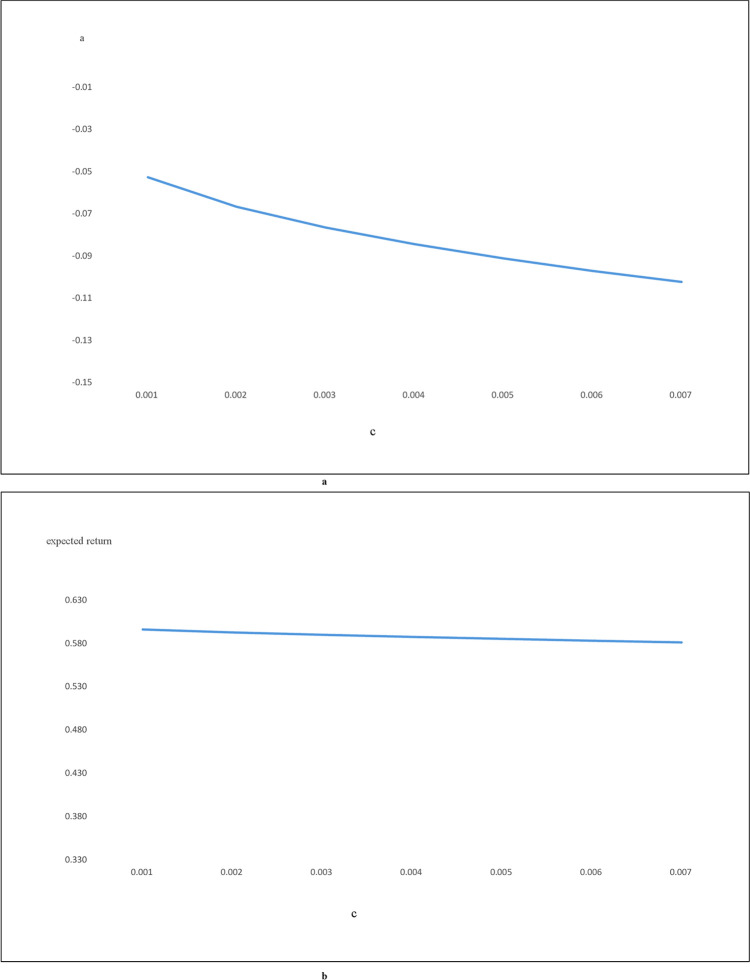
a: c vs a for five-factor model, b: c vs expected return for five-factor model. Fig 7A shows the relationship between transaction cost and a. Fig 7B shows the relationship between transaction cost and the expected return from the optimal trading strategy. The replicating asset for Fig 7 is constructed using the five-factor model.

Figs 6A and 7A plot the relationship between the transaction costs and "a." Figs 6B and 7B plot the relationship between the transaction costs and the expected return of the optimal trading strategy. Fig 6A and 6B correspond to the results of using the Buffett-factor model to construct the replicating asset. Fig 7A and 7B are the results of using the five-factor model. These figures show that higher transaction costs will reduce both the "a" and the expected return of the optimal trading strategy, regardless of which model is used to construct the replicating asset. Figs 6B and 7B, however, show that the expected return of the optimal trading strategy is not that sensitive to transaction costs.

**Fig 8:**
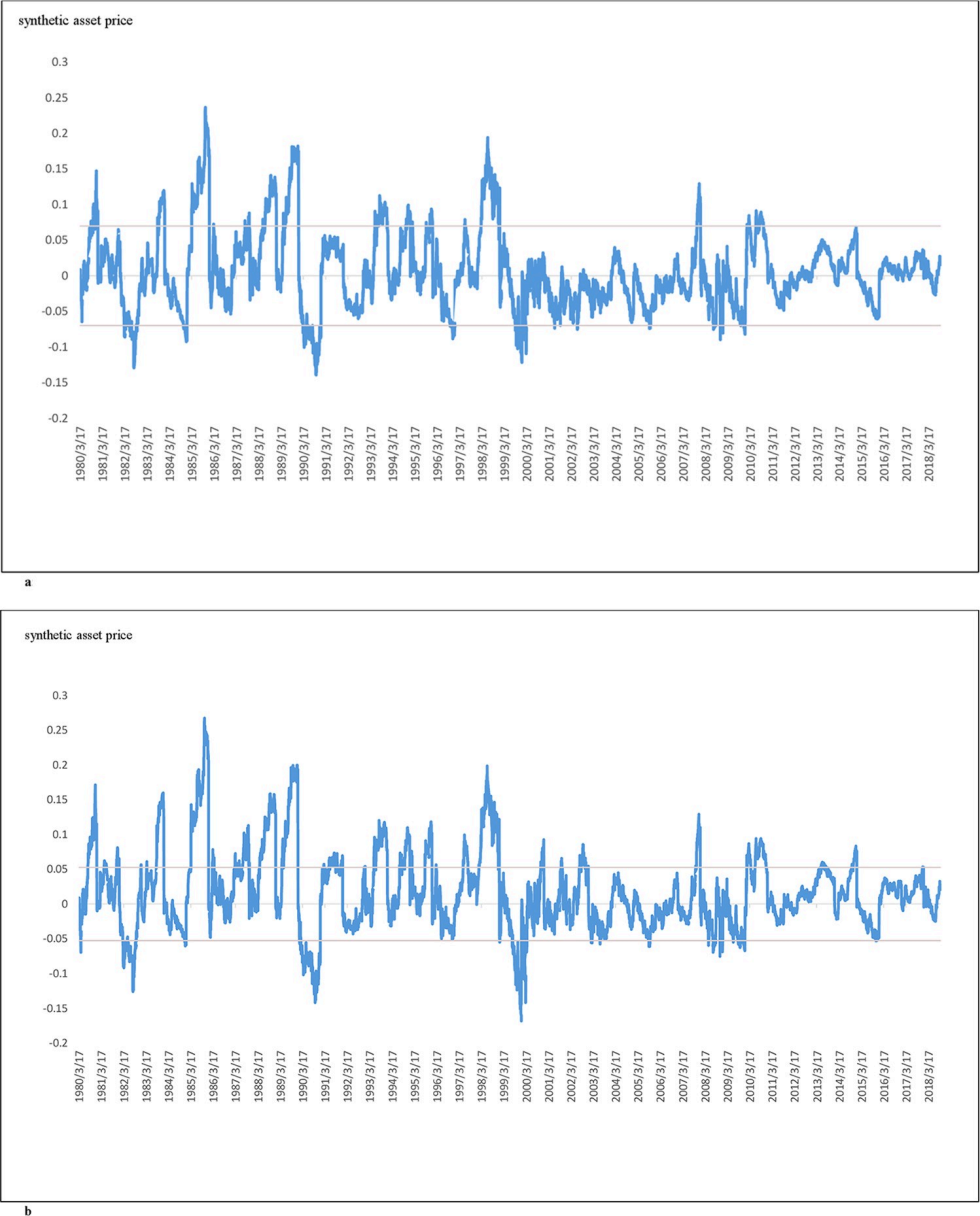
a: The behavior of the synthetic asset formed from Berkshire A stock price and the replicating asset BerkA* when the replicating asset is constructed using the Buffett-factor model, b: The behavior of the synthetic asset formed from Berkshire A stock price and the replicating asset BerkA* when the replicating asset is constructed using the five-factor model. Fig 8A presents the relationship between the synthetic asset price and the entry level "a" and exit level "m." In Fig 8A, the results are computed using the Buffett-factor model to construct the replicating asset. In computing Fig 8A, α = 0.006439, η = 0.05401, and the "a" and "m" are obtained assuming c = 0.001. "c" represents the transaction cost; "a" represents the entry level; "m" is the exit level. Fig 8B presents the relationship between the synthetic asset price and the entry level "a" and exit level "m." In Fig 8B, the results are computed using the five-factor model to construct the replicating asset. In computing Fig 8B, α = 0.01702, η = 0.05748, and the "a" and "m" are obtained assuming c = 0.001. "c" represents the transaction cost; "a" represents the entry level; "m" is the exit level.

Fig 8A and 8B show the relationship between the synthetic asset price and the entry level "a" and exit level "m." Fig 8A provides the results from using the Buffett-factor model to construct the replicating asset. Fig 8B provides the result of using the five-factor model to construct the replicating asset. For Fig 8A, α = 0.006439, η = 0.05401, and the "a" and "m" are obtained assuming c = 0.001. For Fig 8B, α = 0.01702, η = 0.05748, and the "a" and "m" are obtained assuming c = 0.001. "c" represents the transaction cost; "a" represents the entry level; "m" is the exit level.

The results presented show the feasibility of using synthetic assets formed by using the actual stock and a replicating asset constructed from factor models, without the need to find real assets with similar properties for statistical arbitrage. In the experiments, two different factor models were used to replicate the original asset, Berkshire A. Statistical arbitrage applied to the synthetic asset formed from the original Berkshire A and the replicating asset results in arbitrage profits of at least 33% in expected return in Table 1. Our experiments show that the factor model can be used successfully to create replicating assets, which in turn can be combined with the original asset to form a synthetic asset that can be used in pairs trading for optimal statistical arbitrage. We show that the synthetic asset formed from the replicating asset and the original Berkshire A stock can give profitable entry and exit points for statistical arbitrage trading at different transaction costs.

## Robustness tests

**Fig 9:**
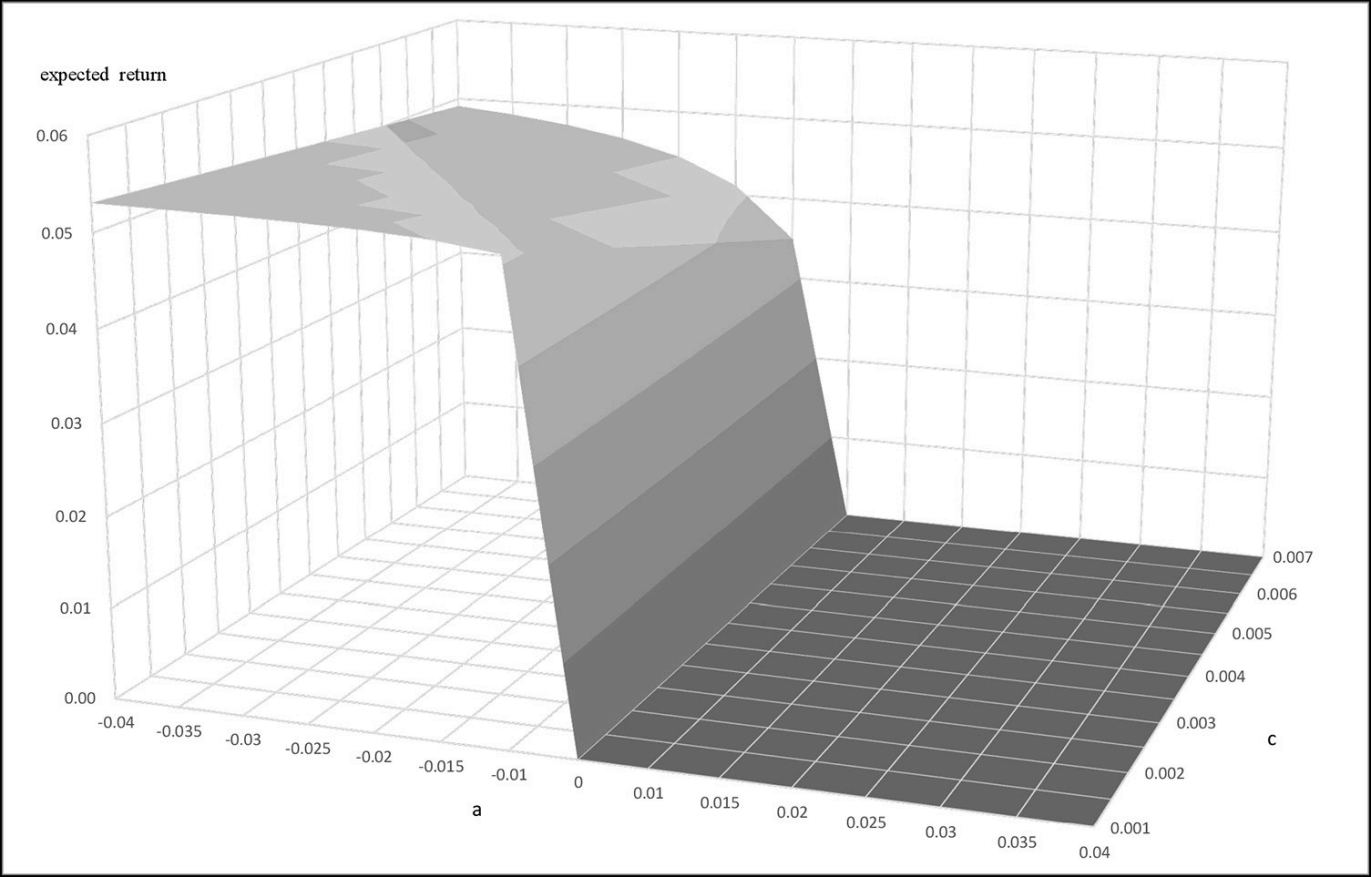
**The relationship of expected return, a, and c for the optimal statistical arbitrage of the S&P500 using the replicating asset constructed by the Buffet-factor model.** This figure presents the relationship of expected return, a, and transaction cost for the optimal statistical arbitrage of the S&P500 using the replicating asset constructed by the Buffett-factor model. This example uses parameters α = 0.008735, η = 0.007170, c = 0.001~0.007.

**Fig 10:**
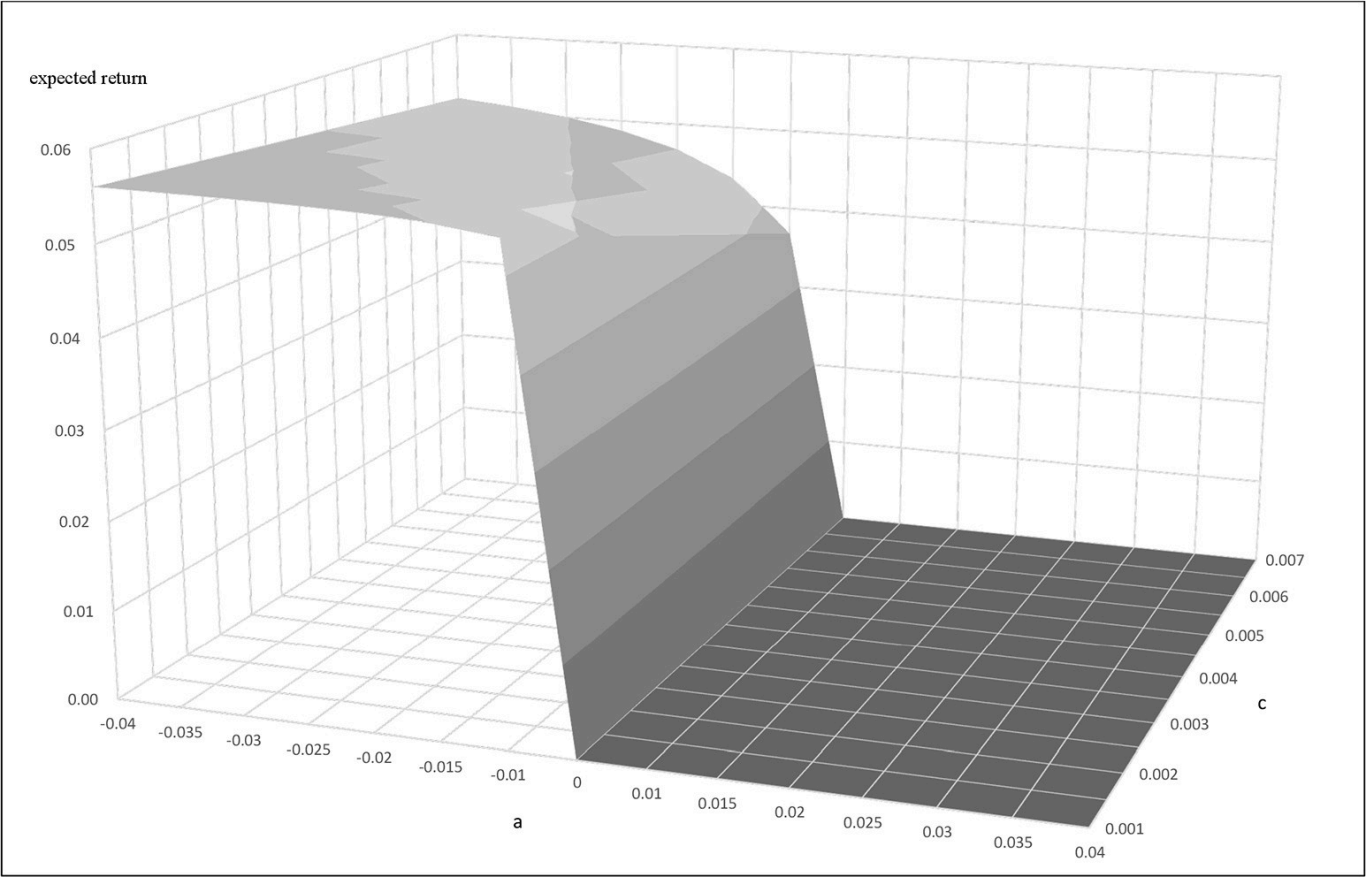
**The relationship of expected return, a, and c for the optimal statistical arbitrage of the S&P500 using the replicating asset constructed by the five-factor model.** This figure presents the relationship of expected return, a, and transaction cost for the optimal statistical arbitrage of the S&P500 using the replicating asset constructed by the five-factor model. This example uses parameters α = 0.009195, η = 0.007369, c = 0.001~0.007.

To test for the robustness of our method, we redo our experiments using the S&P500 index as the target asset to be replicated instead of Berkshire A stock. We check to see if the proposed optimal arbitrage strategy is feasible under the same method. We again use the Buffett-and five-factor model to form the replicating asset. Estimations of parameters were done by using the daily data of the S&P 500 index. The synthetic asset is formed from the replicating asset and the actual S&P 500. We calculate the difference in the log price of the two assets to create the synthetic asset and name it as SP500*. We then calculate the parameters for the Ornstein Uhlenbeck process and get α = 0.008735, η = 0.007170 for Buffett-factor model, and α = 0.009195, η = 0.007369 for five-factor model. Figs 9 and 10 show the trading bands' tests of the S&P500 trading strategy. The two figures show the same overall pattern as those of Figs 4 and 5. The "a" and transaction cost also have an adverse effect on the expected return bands. We use Eq (8) to calculate the optimal "a" value for a given transaction cost, and calculate the expected transaction at the fixed transaction cost and "a". As before, the tests use two different models to construct the replicating asset, the Buffet- and five-factor model. Table 2 shows the optimal strategies at different transaction costs. In Table 2, we find that the expected return of statistical arbitrage of are least 4.8% for the synthetic asset constructed by the two different factor models.

**Table 2:** The expected return of optimal statistical arbitrage of S&P 500 and replicating asset pair.

	Buffett-factor model		five-factor model	
c	a	expected return	a	expected return
0.001	-0.01666	0.0521	-0.01668	0.0550
0.002	-0.02118	0.0512	-0.02120	0.0540
0.003	-0.02443	0.0505	-0.02446	0.0532
0.004	-0.02708	0.0498	-0.02711	0.0525
0.005	-0.02936	0.0492	-0.02939	0.0519
0.006	-0.03138	0.0486	-0.03142	0.0513
0.007	-0.03323	0.0481	-0.03327	0.0507

This table presents the expected return from using the optimal arbitrage strategy under the Ornstein Uhlenbeck process for the synthetic asset formed by the S&P500 and its replicating asset S&P500*. The table lists the expected returns when the replicating asset is constructed using the Buffett- and five-factor model under different transaction costs. The optimal solution for "a" is from Eq (8). For the Buffet-factor model α = 0.008735, η = 0.007170, for the five-factor model α = 0.009195, η = 0.007369. "c" is the transaction cost and "a" represents the entry point.

Tables 1 and 2 show that the obtained returns under statistical arbitrage using the replicating portfolio can depend dramatically on the choice of the asset. There are some possible reasons for this: when conducting optimal statistical arbitrage under our framework, where the synthetic asset is constructed so that it follows an Ornstein Uhlenbeck process, the synthetic asset with a larger variance will likely provide more substantial profits since the synthetic asset by construction should drift back and forth around zero in the long-run, meaning that the synthetic asset with the larger variance will have entry-level "a" and exit level "m" further away from zero, which likely leads to higher profits. In our study, the variance of the synthetic asset formed using Berkshire and its replicating portfolio is larger than the variance of the synthetic asset formed using the S&P 500 and its replicating portfolio; regardless of whether we use the Buffett-factor model to construct the replicating portfolio or the five-factor model to construct the replicating portfolio. Thus, the higher returns for the statistical arbitrage using Berkshire than that of using the S&P 500 is in line with our framework. A possible implication that can be drawn from Table 2 is the possibility that investors can obtain higher returns under the statistical arbitrage framework described in this study if they can find assets when paired with its replicating portfolio, which results in a synthetic asset with high variance.

Next, we perform analyses in terms of Sharpe ratios. Table 3 shows the expected Sharpe ratios from optimal statistical arbitrage of the synthetic asset formed by Berkshire A and its replicating asset BerkA*. Table 4 shows the corresponding Sharpe ratios when the synthetic asset is formed by the S&P500 and its replicating asset S&P500*. As in the previous analyses, we use two different models to construct the replicating asset, the Buffet- and five-factor model. The overall patterns of the Sharpe ratios conform closely to those for the expected returns computed in Tables 1 and 2; and does not affect the main results of this paper, the feasibility of constructing a trading strategy based on statistical arbitrage by constructed a replicating asset.

**Table 3:** The expected Sharpe ratio of optimal statistical arbitrage of Berkshire A and replicating asset pair.

	Buffett-factor model		five-factor model	
c	a	Sharpe ratio	a	Sharpe ratio
0.001	-0.06978	1.952	-0.05274	2.457
0.002	-0.08841	1.950	-0.06678	2.453
0.003	-0.10138	1.948	-0.07663	2.450
0.004	-0.11178	1.947	-0.08452	2.448
0.005	-0.12059	1.945	-0.09122	2.445
0.006	-0.12832	1.944	-0.09712	2.443
0.007	-0.13526	1.942	-0.10241	2.440

This table presents the expected Sharpe ratios from using the optimal arbitrage strategy under the Ornstein Uhlenbeck process for the synthetic asset formed by the Berkshire A stock and its replicating asset BerkA*. The replicating asset is constructed using the Buffett- and five-factor model under different transaction costs. The optimal solution for "a" is from Eq (8). For the Buffet-factor model α = 0.006439, η = 0.05401, for the five-factor model α = 0.01702, η = 0.05748. "c" is the transaction cost and "a" represents the entry point.

**Table 4:** The expected Sharpe ratio of optimal statistical arbitrage of S&P500 and replicating asset pair.

	Buffett-factor model		five-factor model	
c	a	Sharpe ratio	a	Sharpe ratio
0.001	-0.01666	1.669	-0.01668	1.712
0.002	-0.02118	1.660	-0.02120	1.704
0.003	-0.02443	1.653	-0.02446	1.696
0.004	-0.02708	1.646	-0.02711	1.689
0.005	-0.02936	1.639	-0.02939	1.682
0.006	-0.03138	1.633	-0.03142	1.675
0.007	-0.03323	1.627	-0.03327	1.667

This table presents the expected Sharpe ratios from using the optimal arbitrage strategy under the Ornstein Uhlenbeck process for the synthetic asset formed by the S&P500 and its replicating asset S&P500*. The replicating asset is constructed using the Buffett- and five-factor model under different transaction costs. The optimal solution for "a" is from Eq (8). For the Buffet-factor model α = 0.008735, η = 0.007170, for the five-factor model α = 0.009195, η = 0.007369. "c" is the transaction cost and "a" represents the entry point.

## 5. Conclusion

This study provides a novel method of using replicating assets formed by factor models for optimal statistical arbitrage. The synthetic asset formed by the original target asset and the replicating asset follows the Ornstein Uhlenbeck process closely. Our experiments illustrate the viability of forming the synthetic asset from the original target asset and the replicating asset constructed from factor models. The contributions of this study are as follows: first, we show that the factor model can be used to construct a replicating asset that can be paired with the original target asset to perform statistical arbitrage. This method is shown to be executable under different transaction costs assumptions. Since the synthetic asset in this study is formed from the original target asset and the replicating asset constructed from factor models, the method works even when it is not possible to find a suitable tradable asset to pair with the original target asset. Bertram [13] solved the optimal entry level and exit level for statistical arbitrage under the generalized Ornstein Uhlenbeck process. The synthetic asset formed by the original target asset and the replicating asset was shown to satisfy the Bertram [13] optimal statistical arbitrage conditions. Second, we apply the Bertram [13] optimal statistical arbitrage formula to compute the trade length and the arbitrage returns for our replicating asset and target asset pair. These experiments allowed for the analysis of various trading strategies, including the impact of transaction costs. As illustrated in Fig 8, using the factor models to construct the replicating asset for statistical arbitrage allows straightforward determination of the entry levels and exit levels of the arbitrage transactions. We use Berkshire A and S&P 500 to form a synthetic asset with the replicating asset constructed from the factor models. The results of this paper indicate that using the factor models to form the replicating asset and using the optimal solution of the Ornstein Uhlenbeck process to perform statistical arbitrage allows for the determination of entry and exit points under the Bertram [13] optimal statistical arbitrage conditions and is profitable. We believe this approach of using the factor models to construct the replicating asset for statistical arbitrage can be applied to any financial market to gain wealth.
